# Comparison of two methods to clear the airways of critically ill children and adults with COVID-19 infection: a structured summary of a study protocol for a pilot randomized controlled trial

**DOI:** 10.1186/s13063-020-04533-6

**Published:** 2020-07-03

**Authors:** Atsushi Kawaguchi, Gabrielle Bernier, Jacques Lacroix, Saly El Salti, Matthew P. Cheng, Todd C. Lee, Kosar Khwaja, Philippe Jouvet

**Affiliations:** 1 Department of Pediatrics, University of Montreal, CHU Sainte-Justine, 3175 Chemin de Côte Sainte Catherine, Quebec, QB H3T 1C5 Canada; 2 Department of Pediatrics, Children’s Hospital Eastern Ontario, University of Ottawa, Ottawa, Canada; 3 School of Medicine, University of Montreal, Quebec, Canada; 4 Divisions of Infectious Diseases and Medical Microbiology, McGill University Health Centre, Quebec, Canada; 5 McGill Interdisciplinary Initiative in Infection and Immunity, Quebec, Canada; 6 Division of Infectious Diseases, Department of Medicine, McGill University Health Centre, Quebec, Canada; 7 Réseau de Recherche en Santé Respiratoire du Québec, Quebec, Canada

**Keywords:** COVID-19, Randomized controlled trial, Protocol, Chest physiotherapy, Intensive care, Oscillation

## Abstract

**Objectives:**

As there is no treatment for COVID-19 with a proven mortality benefit at this moment in the pandemic, supportive management including mechanical ventilation is the core management in an intensive care unit (ICU). It is a challenge to provide consistent care in this situation, highly demanding and leading to potential staff shortages in ICU. We need to reduce unnecessary exposure of healthcare workers to the virus. This study aims to examine the impact of care using a non-invasive oscillating device (NIOD) for chest physiotherapy in the care of mechanically ventilated patients with COVID-19. In particular, we aim to explore if a NIOD performed by non-specialized personnel is not inferior to the standard chest physiotherapy (CPT) undertaken by physiotherapists caring for patients with COVID-19.

**Trial design:**

A pilot multicenter prospective crossover noninferiority randomized controlled trial.

**Participants:**

All mechanically ventilated patients with COVID-19 admitted to one of the two ICUs, and CPT ordered by the responsible physician. The participants will be recruited from two intensive care units in Canadian Academic Hospitals (one pediatric and one adult ICU).

**Intervention and comparator:**

We will implement NIOD and CPT alternatingly for 3 h apart over 3 h. We will apply a pragmatic design, so that other procedures including hypertonic saline nebulization, intermittent positive pressure ventilation, suctioning (e.g., oral or nasal), or changing the ventilator settings or modality (i.e., increasing positive end-expiratory pressure or changing the nasal mask to total face continuous positive airway pressure) can be provided at the direction of bedside intensivists in charge.

**Main outcomes:**

The primary outcome measurement is the oxygenation level before and after the procedure (SpO_2_/FiO_2_ ratio). For cases with invasive ventilation (i.e., the use of an endotracheal tube to deliver positive pressure) and non-invasive ventilation, we will also document expiratory tidal volume, vital signs, and any related complications such as vomiting, hypoxemia, or unexpected extubation. We will collect the data before, 10 min after, and 30 min after the procedure.

**Randomization:**

The order of the procedures (i.e., NIOD or CPT) will be randomly allocated using manual generated random numbers for each case. Randomization will be carried out by the independent research assistant in the study coordinating center by using opaque sealed envelopes, assigning an equal number of cases to each intervention arm. Stratification will be applied for age (> 18 years or ≤ 18 years of age) and the study sites.

**Blinding (masking):**

No blinding will be performed.

**Numbers to be randomized (sample size):**

We estimate the necessary sample size as 25 for each arm (total 50 cases), with a power of 0.90 and an alpha of 0.05, with a non-inferiority design.

**Trial status:**

The protocol version number 1 was approved on 27 March 2020. Currently, recruitment has not yet started, with the start scheduled by the mid-June 2020 and the end anticipated by December 2020.

**Trial registration:**

ClinicalTrials.gov NCT04361435. Registered on 28 April 2020

**Full protocol:**

The full protocol is attached as an additional file, accessible from the Trials website (Additional File 1). In the interest in expediting dissemination of this material, the familiar formatting has been eliminated; this letter serves as a summary of the key elements of the full protocol.

## Supplementary information

**Additional file 1.** Full Study Protocol.

## Data Availability

Data will be available from the coordinating investigator of this study on reasonable request: Philippe Jouvet, University of Montreal, CHU Sainte-Justine, Department of Pediatrics 3175 Chemin de Côte Sainte Catherine, Montréal, H3T 1C5, QB, Canada Tel: +1-(514)345-4927, Email: philippe.jouvet@umontreal.ca

