# Supplementary material for: Comparison of two methods to clear the airways of critically ill children and adults with COVID-19 infection: a structured summary of a study protocol for a pilot randomized controlled trial
Source: Trials. 2020 Jul 3;21:610. doi: 10.1186/s13063-020-04533-6 (PMC7332538; doi:10.1186/s13063-020-04533-6)
Supplement: Supplementary file 1 — Additional file 1. Full Study Protocol. [file 13063_2020_4533_MOESM1_ESM.pdf]

Comparaison de deux méthodes pour dégager les voies aériennes d'enfant et d'adultes admis aux soins intensifs pour une infection par le COVID-19: Un essai pilote

Proposition de recherche

Short Title
Lung recruitment Device for COVID-19

Principal Investigators;
Philippe Jouvet M.D. Ph.D. MBA 1)

Co-Investigators
Atsushi Kawaguchi MD, PhD[1,2], Gabrielle Bernier[1], Jacques Lacroix MD[1]; Yvon Robert[5]; Lucy Clayton MSc[1]; Matthew P. Cheng MD[3], Todd Lee MD MPH[4], Kosar Khwaja MD[4]

1) University of Montreal, CHU Sainte-Justine, Department of Pediatrics
2) University of Ottawa, Department of Pediatrics, Children's Hospital Eastern Ontario
3) Royal Victoria Hospital
4) Montreal General Hospital
5) Dymedso Inc.
6) Réseau de Recherche en Santé Respiratoire du Québec

Sponsor & Funding Source:
Ministère de l'Économie et Innovation

Contact Investigators:
Philippe Jouvet, M.D. Ph.D. MBA
University of Montreal, CHU Sainte-Justine, Department of Pediatrics
3175 Chemin de Côte Sainte Catherine, Montréal, H3T 1C5, QB, Canada
Tel: +1-(514)345-4927
Email: philippe.jouvet@umontreal.ca

ABSTRACT SUMMARY

INTRODUCTION

As there is no specific cure in the treatment of COVID-19 at this moment of the pandemic, supportive management including mechanical ventilation is the core management in an intensive care unit (ICU). It is a challenge to provide consistent care in this situation of high demand and potential staff shortage in ICU. Also, we need to reduce unnecessary exposure of the providers to the virus. This study aims to examine the impact of care using a non-invasive oscillating device for chest physiotherapy in the care of mechanically ventilated patients with COVID-19.

METHODS

Objective: To explore if a non-invasive oscillatory device (NIOD) performed by non-specialized personnel is not inferior to the standard Chest PhysioTherapy (CPT) done by physiotherapist in the care of COVID-19.
Design: A Pilot Multicenter Prospective Crossover Randomized Study.
Setting: Two Intensive Care Units in a Canadian Academic Hospitals
Patients: All the mechanically ventilated patients admitted to the two ICUs, and CPT ordered by the responsible physician, with COVID-19 infection during the study period.
Procedure: We will implement NIOD and CPT alternatingly for 3 hours apart over 3 hours. We will apply a pragmatic design, so that other procedures including hypertonic saline nebulization, Intermittent Positive Pressure Ventilation (IPPV), suctioning (e.g., oral or nasal), or changing the ventilator settings or modality (i.e., increasing Positive End Expiratory Pressure (PEEP) or changing the nasal mask to total face Continuous Positive Airway Pressure (CPAP)) can be provided at the direction of bedside intensivists in charge. The order of the procedures (i.e. NIOD or CPT) will be randomly allocated for each case.
Measurements and Analyses: The primary outcome measurement is the oxygenation level before and after the procedure (SpO2/FIO2 (SF) ratio). For the cases with Invasive ventilation and non-invasive ventilation, we will also document expiratory tidal volume, vital signs, and any related complications such as vomiting, desaturations, or unexpected extubations. We will collect the data before, 10 minute after, and 30 minutes after he procedure.
Sample Size: We estimate necessary sample size as 25 for each arm (Total 50 cases), with power of 0.90, alfa of 0.05, with the non-inferiority design.

FUTURE CONSIDERATIONS

This randomized pilot study will be considered a running phase if we can/should undertake the RCT which should follow without significant modification of the methods.

1. BACKGROUND

General Information about ICU management for COVID-19

In this catastrophic pandemic of COVID-19, maintaining the provision of healthcare particularly for critical care health care resources has been the key and core matter in each country. Although the proportion of severe or fatal cases may vary by location, 15-30 percent of all hospitalized patients were admitted to the intensive care unit in Italy and China for instance (1-4).

Pneumonia appears to be the most frequent serious manifestation of infection, characterized primarily by fever, sore throat, rhinorrhea cough, and dyspnea. In addition to respiratory symptoms, gastrointestinal symptoms have also been reported (2, 3, 5). The symptomatic infection can range from mild to critical; but, generally, the clinical course seems to have characteristics of slow progression from upper airway to the lower and the other systemic organs such as cardiac muscles leading to severe cardiomyopathy and arrhythmia leading to a circulatory failure.

Acute respiratory distress syndrome (ARDS) is a major complication in patients with severe disease. It has been reported that severe deterioration of oxygenation with ventilatory failure which is less evident. Currently, several medical treatments such as systemic and inhaled steroid, preexisting antiviral agents have been tested as a trial in several regions. Other bedside treatments such as inhale of bronchodilators, prone positioning, and extracorporeal membrane (ECMO). In Italy, as a rescue respiratory management for poor oxygenation and ventilation, regular around-the-clock prone positioning for the ARDS cases have been performed (personal communication, provided in a ESICM (European society of intensive care) webinar meeting on March 22nd).

Staff Shortage & Protection

Protecting the workforce is another critical challenge. Sick leaves or self-isolation has increased already. Caring for infected patients represents a substantial exposure risk for ICU staffs because of high and prolonged exposure to critically ill patients who presumably have higher viral shedding. Severe infections and deaths have occurred among healthcare workers with COVID-19, exerting significant psychosocial stress on the staff. This should be particularly noted for physiotherapists to whom chest physiotherapy can be prescribed more often than usual in this situation of intensive care demand. We should also recognize that only a limited number of physiotherapists are available in the majority of ICUs, which may make it challenging to fulfill the needs of chest physiotherapy (CPT) in the ICUs.

Role of PCT and its Effect

Several scientific papers recently published regarding radiographic findings have presented ground-glass opacities, crazy paving appearance, air space consolidation, broncho-vascular thickening in the lesion, traction bronchiectasis. Another point to note is that the progression of those findings is relatively slow up until 10-14 days from the onset of disease (6-8).

Airway obstruction due to the production of secretion particularly with respiratory muscle weakness in critically ill is a major problem in clinical management. CPT and invasive positive percussion ventilation (IPPV) have been recognized as to encourage dislodging the secretions; nonetheless, the tolerance to the procedure and its efficiency have not been proved to be sufficient.

CPT has been proposed to assist in the clearance of tracheobronchial secretions. The goal is to clear the mechanical airway obstruction, reduce airway resistance, enhance gas exchange, and reduce the work of breathing eventually. Techniques such as the conventional CPT with chest percussion and vibration, chest shaking and directed coughing may help to mobilize secretions towards the trachea and trigger coughing that could help to remove the secretions.

Although CPT still has been playing an important role in an intensive care as an alternative practice of airway management, a recent systematic review reported that there is no sufficient evidence for us to provide CPT in routine practice for the patients with respiratory distress or failure. At the same time, we need to be aware of the significant heterogeneity of the results among the evidence and inconsistency of the procedure techniques utilized (9-13).

Extra-thoracic non-invasive oscillating devices (NIOD) are designed to interrupt the expiratory airflow, vibrating at variable frequencies and intensities as set by the operator to ensure the individual’s comfort and associated concordance. A combination of strong and weak coupling to the curved chest wall can transmit vibrations to both deep and shallow tissues. Peristaltic action due to longitudinal waves could provide better clearance. Generally, NIOD requires less patient cooperation and could be used without interruptions such as due to coughing or suctioning of secretion. Frequencer® (Dymedso, Montreal Canada) is a NIOD distinguishing itself by being able to locally target the affected area, instead of influencing entirely the patients’ lungs. The safety has been attested particularly in patients with cystic fibrosis. In addition, this NIOD can be easily administered with simple instruction by patients themselves, in other words, by non-specialized personnel such as respiratory therapists or registered nurses as an alternative procedure of regular CPT done by a physiotherapist (14, 15).

This proposed study is to compare the effectiveness of two methods of mobilizing secretions in ICU patients for respiratory distress with COVID-19 infection: NIOD performed by non-specialized personnel (respiratory therapist, nurse, physicians) versus standard CPT performed by a physiotherapist.

2. OBJECTIVES & RESEARCH QUESTIONS

The proposed study has specific objectives and research questions. Our population of interest is all the patients mechanically ventilated for whom CPT is prescribed to aim airway clearance in the participating ICUs. Patients can be either invasively mechanically ventilated or non-invasively mechanically ventilated (MV) such as with high flow nasal therapy or mask positive pressure ventilation.

Objectives

This project has 3 specific objectives:

1) To explore if there is a possibility that the NIOD performed by non-specialized personnel is not inferior to the standard CPT done by physiotherapist in the care of COVID-19.
2) To verify the feasibility and tolerance of NIOD in the mechanically ventilated patients
3) To examine the impact of NIOD and CPT on oxygenation (primary outcome: SpO_2 / FiO_2 ratio).

Research questions to be asked

1. Is the NIOD safe and well-tolerated to be applied to the critically ill patients with COVID-19?
2. Is the NIOD not inferior to standard CPT performed by physiotherapist with respect to the effect of oxygenation in critically ill COVID-19 patients?
3. What kinds of physiological positive effects can the NIOD (and CPT) provide on critically ill patients with COVID-19?

3. SIGNIFICANCE

This study will provide valuable knowledge and improvements in regard to secretion management in critically ill patients. It will not only produce evidence for our current practice but also contribute to more efficient health care practice and resource utilization (i.e., physiotherapist) and improved quality of care.

To be specific, NIOD could improve the respiratory management of patients with COVID-19, especially since NIOD could be performed more often than the standard CPT by physiotherapist. The findings acquired in this study can be generalizable to other hospitals and cohorts in ICUs. Ultimately, this could reduce the impact of COVID-19 on the health system. Also, probably most importantly, we could reduce and optimize the exposure of physiotherapists to COVID-19, which leads to a better working conditions in ICUs.

4. METHODS

4.1 Study Design

We will adopt a prospective crossover non-inferiority randomized design.

4.2 Setting

Two Intensive Care Units in CHU Sainte Justine (Pediatric ICU) and Montreal General (Adult ICU) hospital.

4.3 Screening of Eligible Patients and Informed Consent Procedure.

When there is a potentially eligible patient, a research assistant (and/or student) will be notified in 24/7 during the study period. We will obtain the informed consent (IC) from patients or parents/guardians as soon as possible from the identification of the patients. The research assistant will obtain the IC using an informed consent from.

4.4 Eligible Patients

4.4.1 Inclusion Criteria

All the patients admitted to the ICUs during the study period will be screened. We will not set any restriction regarding the timing of prescription of CPT (i.e., length of ICU stay before screening) for the screening. We will include only if CPT is expected to be used as a management at least for the next 24 hours in the ICUs from the time of inclusion. For instance, if CPT will be expected to be discontinued from the management in a day, we will exclude them from the inclusion. CPT can be prescribed for airway clearance with any etiology such as atelectasis at the directions of bedside intensivists in charge on the study date.

<u>Specific Insclusion criteria</u>

- Patients diagnosed as COVID-19
- Patents strongly suspected its infection and the result of virological testing is pending as of screenig.
- Patients with any type of mechanical ventilation (i.e., Invasive, non-invasive, high flow nasal therapy)
- Patients is on standard oxygen by mask or nasal cannula and the FiO_2 provided can be measurebale.
- Patienst who is on respiratory monitoring at least SpO_2 can be continously measurable.

4.4.2 Exclusion Criteria

- CPT order will (or is expected to) be discontinued within 12 hours from the inclusion timing. This may include potential discharge from the unit.
- CPT is not ordered for airway clearance.
- SpO_2 is not stable (SpO_2=<80%) with more than 0.60 of F_IO_2 for the ventilated patients including patients on NIV, at least for previous 1 hour from the screening.
- SpO_2 is not stable (SpO_2=<80%) with more than 0.60 of F_IO_2 for the patients on HFNC, at least for previous 1 hour from the screening.
- Bradycardia (HR<50bpm) at any interventions at least for 24 hours prior to the screening.
- Patients with known pneumothorax, osteomyelitis at ICU admission.
- Thoracotomy within 1 month from the admission date.
- Known recent/unhealed rib fractures.
- Known skin injury of chest wall.
- No obtain of IC.
- Brain death or vegetated states.

4.5 Outcomes

Primary Outcome

- SpO_2/FIO_2 Ratio (before the procedure and 10 minutes from the end of the procedure) (16-21)

Secondary Outcomes

- Changes in Vital sign values including blood pressures, heart rates, respiratory rate, temperature, neurological status, work of breathing.
- Changes in expiratory tidal lung volume.
- Changes in $EtCO_2$ values and blood gas parameters.
- Clinical Respiratory severity scores.

4.6 Intervention:

Each subject will receive the NIOD (i.e., Frequencer) and the standardized CPT for this study.

4.6.1 Suctioning and Other Interventions

As per the each ICU's protocol, subjects will have suctioning of secretion as needed. Any other potential interventions such as nebulization of hypertonic saline and bronchodilator, and that with metered-dose inhaler (MDI) can be given without any restrictions.

4.6.2 Positioning

All the procedure (i.e., NIOD and CPT) will be applied with any positioning such as sitting and prone positions. Caregivers can also change the positioning as needed during the procedures. All the outcome parameters should be measured (before and after the procedures) with the same positioning.

4.6.3 Detailed Procedure

NIOD will be implemented on four different parts of the chest walls, 3 minutes for each part and 12 minutes in total per each session. Left and right front and posterior chest walls will be stimulated, particularly, on the anterior chest, intercostal spaces 1-2 above nipple line and lateral side of the mid-clavicular line 1-2 below intercostal spaces. The intensity of the NIOD can be selected between 80-100%, which is pre-specified on the machine. The step-by-step procedure is described in the **Appendix**.

4.6.4 Selection of the Membrane

There are several selections of the membrane available. We will use the membrane according to the manual provided by the manufacture.

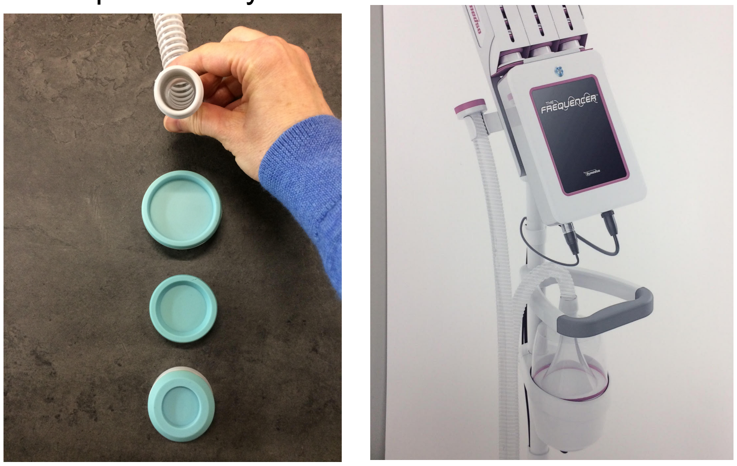

Figure1a Transducers and new prototype NIOD (Frequencer)

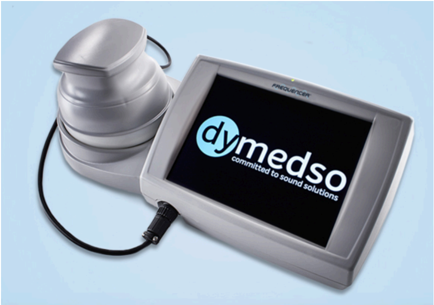

Figure1b Transducers and NIOD (Frequencer) for adults

4.6.5 Standardization of the Procedure

The procedure using the NIOD will be standardized. We will use a video clip (i.e., YouTube) and will ask each caregiver to view at least once before the first procedure of the day. If the single caregiver will perform a NIOD multiple times in a day on the identical patient(s), we will allow them to perform the NIOD without watching the video repeatedly.

4.6.6 CPT

CPT will be defined as an assistant strike to the chest wall repeatedly with a cupped hand in specific places. CPT will be performed by a physiotherapist as per protocol in each institution.

4.7 Schematic Overview of the Protocol

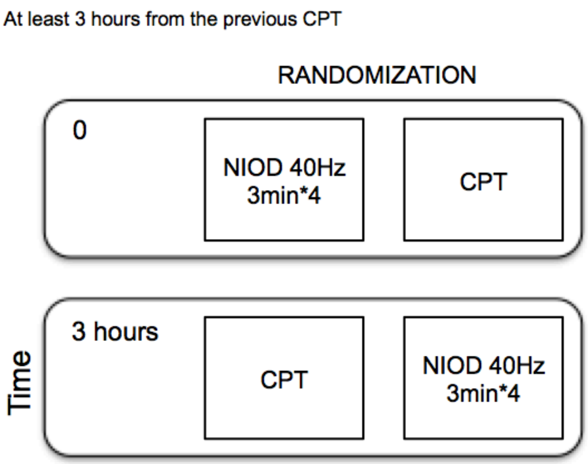

Figure 2 Overview of the procedures

Note: 60 Hz will be applied for children

4.8 Detailed Protocol

Patients satisfying the eligibility criteria will have their baseline data collected prior to the intervention.

Randomization

Patients will be randomized into one of the 2 arms (NIOD first or CPT first). In the existing studies targeting adults, 20-60Hz are most widely used (personal communication with Dymedso Inc.). We will apply 40Hz. Randomization will be carried out by the independent research assistant in CHU Sainte Justine (CHUSJ). Random allocation will be generated by an independent investigator in an equal number assigned to each intervention. Stratification will be applied for age (>18 years or =<18 years of age) and sites (CHUSJ and General Hospital) (Figure2).

Timing of the Data Measurement

The data measured in mechanical ventilation and patients monitor will be collected 1) right before the intervention being initiated, 2) 10 minutes after the end of the intervention, and 3) 30 min after each intervention finished. We will manually record the following variables for the three timings (4.9.1). Vital sign values including $EtCO_2$ waveforms from the beginning of the intervention till 30 min after the intervention will be extracted from the Electronic Medical Records (i.e., every minutes) for the cohorts in CHUSJ.

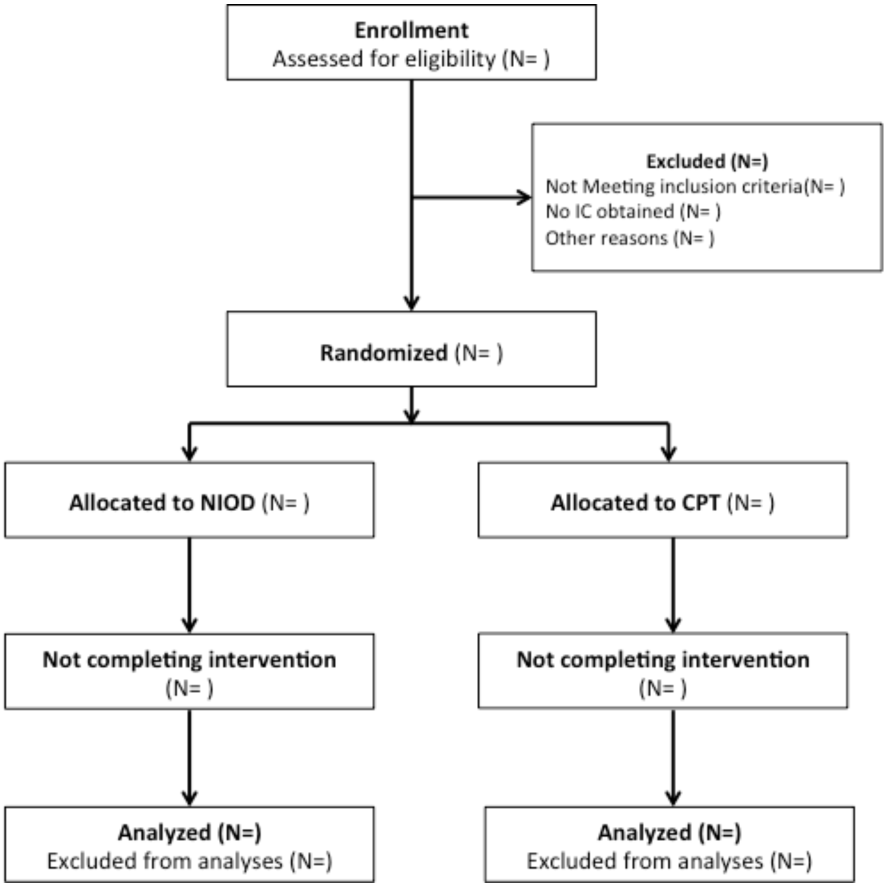

Figure 3 Planned CONSORT diagram

4.9 Collection of Data for Each Patient

4.9.1 Data Elements to be Collected

- Chronologically number will be applied for each study subject.

Demographics

- Gender
- Age (DOB)
- ICU Admission date and Time
- Date and time of informed consent
- Date and Time study procedure performed
- Weight (kg)
- Chest radiograph interpretation prior to study if obtainable
- Diagnoses based on ICD-10 codes including chronic and acute diseases.
- Condition of COVID-19 (confirmed or result pending)
- Results of concomitant infections (viral panel, blood, secretion, urine, and stool)

Measured Outcome

These items will be recorded i) Before, ii) 10 minutes after each procedure, and iii) 30 minutes from the procedure.

- SpO_2/FIO_2 (SF) Ratio. FIO_2 will be measured from the ventilators (Invasive, NIV, HFNC).
- Vital signs including blood pressures, heart rates, respiratory rate, temperature, neurological status (e.g., Glasgow Coma Score), work of breathing (i.e., mild, moderate, and severe). Signs of increasing work of breathing will be evaluated based on retraction (intercostal, suprasternal, costal margin, paradoxical abdominal breathing) and accessory muscle use (nasal flaring, sternomastoid contraction, and forward posture). When the vital sign values are not stable and fluctuating during the measurement, most reliable values considering median will be recorded.
- Expiratory Tidal volume, measured PEEP, and PIP when patients are on InMV or NIV.
- Value of $EtCO_2$.
- Severity of disease (APACHE, SOFA for adults and PELOD 2 for children)
- Respiratory severity score (Wood modified score).
- Potential complications including any significant events during the procedure will be recorded.
- The blood gas findings before and after the procedure and the timing of the sample being taken will be recorded when they are available.
- Any free comments from the care providers will be documented regarding the procedure.

4.10 Sample Size Calculation

A trial aims to determine whether NIOD will be able to provide an improvement of oxygenation not inferior than standard CPT. We estimate mean improvement of SF ratio is 30 with variance of 10 in CPT. It was claimed to be clinically non-inferior if effect of NIOD was 20% lower than the standard CPT. The largest clinically acceptable difference of 6 (30*0.20) is declared that NIOD is non-inferior compares with CPT. Type I, II error, and ratio are set at 0.05, 0.1, and 1:1. With this, we require 32 cases in total (16 cases per group). Considering this, we will collect 25 cases per group (16-21).

4.11 Protocol Violation

Protocol violation in eligibility is defined as when a patient was randomized, but does not follow the protocol. These patients will be excluded from the analyses. Patients who withdraw their consent after randomisation will be excluded from the analyses. The number of excluded patients after randomisation will be displayed in the CONSORT flow diagram (Fig3). Patients assigned to both arm receive the standard ICU care in each center.

4.12 Statistical Analyses

The main analysis will be performed according the intention-to-treat (ITT) principle. In the ITT analysis all patients are analysed according to their initially assigned study arm at baseline, regardless of adherence to study protocol. Patient who withdrew consent or patients with a protocol violation concerning eligibility are excluded from ITT analyses. Differences in patient characteristics between patients lost to follow-up and included patients will be assessed. In view of the non-inferiority study design, per protocol (PP) analyses will also be performed. All subjects from the ITT population without protocol violations and deviations regarding treatment will be included in the PP population. We will also repeat the analyses with logistic regression to take into account that randomisation was stratified by the participating center and age (>18 or =<18 years of age). Non-inferiority will be established if the lower limit of the one-sided 95% confidence for the mean of SF ratio for CPT group relative to that of patients with NIOD exceeds the critical value corresponding to the absolute 5% margin below the observed proportion under usual care. All statistical analyses will be conducted with Stata version 13® (Stata Corp LP, TX USA) or other statistical software(s).

5. ETHICAL CONSIDERATIONS

5.1 Overview

The device and membrane for adults applied has been certified and approved by Health Canada and commercially available in Canada (Fig1a). Although the prototype transducer for infants (Fig1b) has not been tested on patients as a large trial, the mechanism of the pressure delivery and the intensity of the delivering pressure/ oscillation should be similar as compared to the existing model for children and adult patients. The devices have been approved by Health Canada. We believe there should be no excessive harm as compared to the current standard CPT. Patients will also receive standard CPT during the study period, which is widely accepted as a practice for the airway clearance in the ICUs.

This study will be approved by the Health Research Ethics Board of University of Montreal, Canada. The study protocol will be registered into https://clinicaltrials.gov/ once it is approved by the local IRB.

5.2 Risk Minimization and Data and Safety Monitoring Board (DSMB)

It has shown that NIOD was feasible and well tolerated in adult and relatively older children. For this study, another DSMB will be in place to independently supervise any potential side effects. It will include Francois Proulx MD and Tse Man Sze MD. The DSMB will follow the study completion and review any significant potential complications

related to the intervention with NIOD. Any occurrence of pneumothorax, need for unplanned intubation, life-threatening event, during or in the 2 hours following the study, will be declared to and reviewed by the DSMB, which will independently decide on the need to stop or continue the study.

6. RESEARCH TEAM

The research team includes critical care specialists (PJ, AK, and KK) and infectious disease specialist (MC and TL), industrial partner (YR) and methodologist (AK). The knowledge translation will be applied throughout the study among the research team. PJ in CHU Sainte Justine (CHUSJ) is the Principal Investigators (PI) of this project; he will coordinate the project team and the implementation of the project's findings into the clinical community. PJ, AK and KK will provide the team with advice on methodologies and lead in the dissemination and application of knowledge. The team will have a regular bi-weekly video meeting.

7. KNOWLEDGE TRANSLATIONS

The goal of this pilot trial is to increase knowledge and change practice promptly in health care professionals for the care of COVID-19. As soon as the analyses are finished, we will disseminate our findings through peer-reviewed publications and conference presentations in critical care fields (e.g., *Intensive Care Med*) .

8. MILSTONES

- Discussion already made with the industrial partner (Dymedso Inc.) who is said to be ready to supply NIODs by April 02, 2020.
- Study Protocol and submission for the two participating sites (CHUSJ and McGill):
- Training of workers in the two sites: by April 5, 2020.
- Contractual agreement between players (sites, companies): by April 5, 2020 if possible.
- Beginning and end of the pilot randomized study (n=50): from mid-April to end of April.
- Data analysis: by end of April 2020.
- Results sent to government authorities in May
- We will take the steps required to undertake a final RCT if the results of the pilot study suggest that this would be relevant.

Appendix Detailed Mechanism of the NIOD

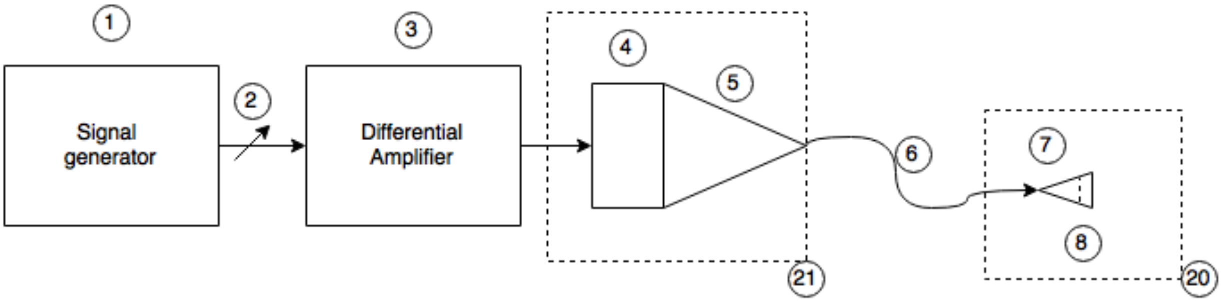

1. Signal generator used to generate a sine wave from 20 to 65Hz with amplitude from 0 to 100%
2. Calibration
3. Differential amplifier
4. Electric to acoustic converter
5. Sound wave reduction flute
6. Logarithmic sound wave flute to reduce the diameter of the vibration chamber from 4” to 1” allowing a small tube to be connected.
7. Flexible tube
8. This tube was selected with care to optimize the wave transmission and minimize as much as possible the attenuations that could reduce the output at the adapter side. The tube is very thin and light, but is assembled on a stronger spiral material on its outer layer to minimize the obstacles inside the tube and augment its resistance to manipulations.
9. Acoustic amplification chamber
10. Logarithmic sound wave flute to increase the internal diameter from 1” to 2”. This part allows the more space for the air exchange that is needed to create the vibration of the membrane
11. Vibration membrane
12. A thin membrane made of silicon that vibrates to the original frequency output by the transducer. The membrane is allowed to move freely without touching the skin since it is recessed on the adapter.
13. Adapter converting a pressure wave into a vibration (motion and sound)
14. Transducer transforming the electrical signal into an acoustic signal
15. The combination of #20 & #21 is air-tight to optimize the sound and vibration by minimizing losses and attenuations

REFERENCES

1. Grasselli G, Pesenti A, Cecconi M. Critical Care Utilization for the COVID-19 Outbreak in Lombardy, Italy: Early Experience and Forecast During an Emergency Response. JAMA. 2020.
2. Huang C, Wang Y, Li X, Ren L, Zhao J, Hu Y, et al. Clinical features of patients infected with 2019 novel coronavirus in Wuhan, China. Lancet. 2020;395(10223):497-506.
3. Wang D, Hu B, Hu C, Zhu F, Liu X, Zhang J, et al. Clinical Characteristics of 138 Hospitalized Patients With 2019 Novel Coronavirus-Infected Pneumonia in Wuhan, China. JAMA. 2020.
4. Wu Z, McGoogan JM. Characteristics of and Important Lessons From the Coronavirus Disease 2019 (COVID-19) Outbreak in China: Summary of a Report of 72314 Cases From the Chinese Center for Disease Control and Prevention. JAMA. 2020.
5. Yang X, Yu Y, Xu J, Shu H, Xia J, Liu H, et al. Clinical course and outcomes of critically ill patients with SARS-CoV-2 pneumonia in Wuhan, China: a single-centered, retrospective, observational study. Lancet Respir Med. 2020.
6. Kanne JP, Little BP, Chung JH, Elicker BM, Ketai LH. Essentials for Radiologists on COVID-19: An Update-Radiology Scientific Expert Panel. Radiology. 2020:200527.
7. Pan F, Ye T, Sun P, Gui S, Liang B, Li L, et al. Time Course of Lung Changes On Chest CT During Recovery From 2019 Novel Coronavirus (COVID-19) Pneumonia. Radiology. 2020:200370.
8. Shi H, Han X, Jiang N, Cao Y, Alwalid O, Gu J, et al. Radiological findings from 81 patients with COVID-19 pneumonia in Wuhan, China: a descriptive study. Lancet Infect Dis. 2020.
9. Roque i Figuls M, Gine-Garriga M, Granados Rugeles C, Perrotta C, Vilaro J. Chest physiotherapy for acute bronchiolitis in paediatric patients between 0 and 24 months old. Cochrane Database Syst Rev. 2016;2:CD004873.
10. Chaves GS, Freitas DA, Santino TA, Nogueira PAM, Fregonezi GA, Mendonca KM. Chest physiotherapy for pneumonia in children. Cochrane Database Syst Rev. 2019;1:CD010277.
11. Hough JL, Flenady V, Johnston L, Woodgate PG. Chest physiotherapy for reducing respiratory morbidity in infants requiring ventilatory support. Cochrane Database Syst Rev. 2008(3):CD006445.
12. Lauwers E, Ides K, Van Hoorenbeeck K, Verhulst S. The effect of intrapulmonary percussive ventilation in pediatric patients: A systematic review. Pediatr Pulmonol. 2018;53(11):1463-74.
13. Ferreira LL, Valenti VE, Vanderlei LC. Chest physiotherapy on intracranial pressure of critically ill patients admitted to the intensive care unit: a systematic review. Rev Bras Ter Intensiva. 2013;25(4):327-33.
14. Dalma Schieppati ReG, Federico Galli, Marco Giulio Rigamonti, Marta, Stucchi DCB. Influence of operating frequency and amplitude in the Frequencer™ to treat cystic fibrosis.

15. Cantin AM, Bacon M, Berthiaume Y. Mechanical airway clearance using the frequencer electro-acoustical transducer in cystic fibrosis. Clin Invest Med. 2006;29(3):159-65.
16. Adams JY, Rogers AJ, Schuler A, Marelich GP, Fresco JM, Taylor SL, et al. Association Between Peripheral Blood Oxygen Saturation (SpO2)/Fraction of Inspired Oxygen (FiO2) Ratio Time at Risk and Hospital Mortality in Mechanically Ventilated Patients. Perm J. 2020;24.
17. Bilan N, Dastranji A, Ghalehgolab Behbahani A. Comparison of the spo2/fio2 ratio and the pao2/fio2 ratio in patients with acute lung injury or acute respiratory distress syndrome. J Cardiovasc Thorac Res. 2015;7(1):28-31.
18. Ghazal S, Sauthier M, Brossier D, Bouachir W, Jouvet PA, Noumeir R. Using machine learning models to predict oxygen saturation following ventilator support adjustment in critically ill children: A single center pilot study. PLoS One. 2019;14(2):e0198921.
19. Koyauchi T, Yasui H, Enomoto N, Hasegawa H, Hozumi H, Suzuki Y, et al. Pulse oximetric saturation to fraction of inspired oxygen (SpO2/FIO2) ratio 24 hours after high-flow nasal cannula (HFNC) initiation is a good predictor of HFNC therapy in patients with acute exacerbation of interstitial lung disease. Ther Adv Respir Dis. 2020;14:1753466620906327.
20. Kwack WG, Lee DS, Min H, Choi YY, Yun M, Kim Y, et al. Evaluation of the SpO2/FiO2 ratio as a predictor of intensive care unit transfers in respiratory ward patients for whom the rapid response system has been activated. PLoS One. 2018;13(7):e0201632.
21. Wiedermann FJ, Stichlberger M, Glodny B. ARDS diagnosed by SpO2/FiO2 ratio compared with PaO2/FiO2 ratio: the role as a diagnostic tool for early enrolment into clinical trials. Open Med (Wars). 2016;11(1):297.
